# Soy foods have low glycemic and insulin response indices in normal weight subjects

**DOI:** 10.1186/1475-2891-5-35

**Published:** 2006-12-27

**Authors:** Robert M Blair, EC Henley, Aaron Tabor

**Affiliations:** 1 Physicians Pharmaceuticals, Inc., 1031 E. Mountain St., Building 302, Kernersville, NC 27284, USA

## Abstract

**Background:**

Foods with a low glycemic index (GI) may provide a variety of health benefits. The objective of the present study was to measure the GI and insulin index (II) of select soy foods.

**Methods:**

The study was conducted in two parts with low-carbohydrate products being tested separately. In Experiment 1, subjects averaged 23.2 years of age with BMI = 22.0 kg/m^2^, while subjects in Experiment 2 averaged 23.9 years of age with BMI = 21.6 kg/m^2^. The reference (glucose) and test foods were served in portions containing 10 g of carbohydrates in Experiment 1 (two test foods) and 25 g of carbohydrates in Experiment 2 (four test foods). Subjects consumed the reference food twice and each test food once. For each test, subjects were instructed to consume a fixed portion of the reference food or test food together with 250 g of water within 12 min. Blood samples were collected before each test and at 15, 30, 45, 60, 90, and 120 min after consumption of reference or test foods to quantify glucose and insulin. Two-hour blood glucose and plasma insulin curves were constructed and areas under the curves were calculated. GI and II values for each subject and test food were calculated.

**Results:**

In Experiment 1, both low-carbohydrate soy foods were shown to have significantly (P < 0.05) lower GI and II values than the reference food. In Experiment 2, three of the four test foods had significantly (P < 0.05) lower GI and II values than the reference food.

**Conclusion:**

All but one of the soy foods tested had a low GI, suggesting that soy foods may be an appropriate part of diets intended to improve control of blood glucose and insulin levels.

## Background

The glycemic index (GI) was first developed by Jenkins and colleagues [1] as a new method of classifying foods based on the blood glucose response after food consumption. The GI value of a food is a percentage of the 2-hour area under the blood glucose response curve of a reference food, typically glucose [2]. Since the GI is determined for a particular quantity of carbohydrates in the food being tested and since the actual amount of carbohydrates consumed in a meal or snack varies greatly, the GI concept was expanded to include the concept of glycemic load (GL). The GL is determined by multiplying the GI of a food by the grams of carbohydrates in a serving. The GL value incorporates the amount of digestible carbohydrates in a serving in order to better gauge the impact of a meal or snack on postprandial glucose response [3,4].

It has been reported that a high GI diet may have adverse health consequences by increasing the risk for chronic disease [5,6]. Recent evidence suggests that high GI/GL diets may increase the risk for cardiovascular disease (CVD) [7-9] and type 2 diabetes [3,4,10,11]. A high GI diet may increase the risk for chronic disease through the stimulation of hyperglycemia and hyperinsulinemia [6].

In contrast, a low GI diet has been reported to have health benefits [5,6,12,13]. A low GI diet has been shown to improve glycemic control [14-17], aid in weight loss [18,19], and reduce some CVD risk factors [9,14,20-23].

To date, only about 30 – 40 soy foods have been assessed for their GI/GL values [1,24-27]. The objective of the current study was to determine the GI and insulin index (II) values of select soy food products (bars, drinks, pasta, and chips) currently available on the U.S. and international markets.

## Methods

The current study was conducted using internationally recognized GI methodology [28-30]. The experimental procedures used in this study were in accordance with international standards for conducting ethical research with humans and were approved by the Human Research Ethics Committee of Sydney University of Australia where the study was conducted by contract.

A search of the literature using the National Library of Medicine's PubMed search engine showed a paucity of literature on the determination of the glycemic index of soy foods. A search of "soy AND glycemic index" yielded 5 hits; a search of "soy AND glycemic load" yielded 2 hits; a search of "soy AND glycemic" yielded 14 hits and a search of "isoflavone AND glycemic" yielded 5 hits. However, none of the papers found measured the glycemic index or glycemic load of soy foods. A search of the international table of glycemic index and glycemic load values [26] showed that the glycemic index of several soy foods has been measured, but only a few of these have been reported as independent publications.

### Study Subjects

For both experiments, 10 healthy, non-smoking subjects, were recruited from the staff and student population of the University of Sydney. Exclusion criteria included being overweight, dieting, impaired glucose tolerance, illness or food allergy, or regular use of prescription medication (other than contraceptive medication). All study participants gave written informed consent before participating in this study.

In Experiment 1 the 10 subjects (two females, eight males) had a mean age of 23.2 years (19.9–25.7 years) and a mean body mass index (BMI) score of 22.0 kg/m^2 ^(absolute range = 19.4–25.4 kg/m^2^). In Experiment 2 the 10 subjects (four females, six males) had a mean age of 23.9 years (20.3–26.9 years) and a mean BMI score of 21.6 kg/m^2 ^(absolute range = 19.5–25.8 kg/m^2^). Four subjects participated in both experiments.

### Composition of Test Foods

In Experiment 1, the reference and two low-carbohydrate test foods (products with ≤6 g net carbohydrates (available carbohydrates); net carbohydrates = total carbohydrates – sugar alcohols – fiber) were served to subjects in portions containing 10 g of digestible carbohydrate. In Experiment 2, the reference and four test foods were served to subjects in portions containing 25 g of available carbohydrate. Glucose (Glucodin^® ^powder, Boots Health Care Company, North Ryde, NSW Australia) dissolved in water was the reference food. Mass and nutrient contents of the reference and test foods (Revival Soy^® ^from Physicians Pharmaceuticals, Inc., Kernersville, NC, USA) are listed in Tables 1 and 2.

**Table 1:** Mass and nutrient contents of the test portions of the glucose reference and the two test foods in Experiment 1.

Test Food	Portion Size (g)^1^	Energy (kJ [Cal]) {kj/100 g of product}	Protein (g)^2 ^{g/100 g of product}	Fat (g) {g/100 g of product}	Available Carbohydrate (g) {g/100 g of product}	Sugars (g) {g/100 g of product}	Fiber (g) {g/100 g of product}
Glucose (Reference Food)	10.0 g glucose 250 g water	160 [38.2]	0.0	0.0	10.0	10.0	0.0
Chocolate Raspberry Zing™ bar	150.0	2205 [526.7] {1,470}	45.0 {30.4}	12.5 {8.4}	10.0 {7.7}	0.0 {0.0}	< 2.0 {1.3}
Chocolate Daydream™ shake – sucralose	70.0 g powder 500.0 g water	1092 [260.8] {1,551}	40.0 {57.1}	5.0 {7.1}	10.0 {14.3}	2.0 {2.9}	4.0 {5.7}

^1^The amount of water listed for some products is the amount of water needed to prepare that product. This volume is in addition to the 250 g of water consumed with every product as part of the test methodology.

^2^All of the protein in the products above is derived from soy. Each Chocolate Raspberry Zing™ bar contained 21 g soy protein. Each Chocolate Daydream™ shake contained 20 g soy protein.

**Table 2:** Mass and nutrient contents of the test portions of the glucose reference and the four test foods in Experiment 2.

Test Food	Portion Size (g)	Energy (kJ [Cal]) {kj/100 g of product}	Protein (g)* {g/100 g of product}	Fat (g) {g/100 g of product}	Available Carbohydrate (g) {g/100 g of product}	Sugars (g) {g/100 g of product}	Fiber (g) {g/100 g of product}
Glucose (Reference Food)	25.0 g glucose 250 g water	400 [95.5]	0.0	0.0	25.0	25.0	0.0
Peanut Butter Chocolate Pal™ bar	48.4	813 [194.2] {1,671}	12.9 {26.7}	4.8 {10.0}	25.0 {51.7}	14.5 {30.0}	0.8 {1.7}
Chocolate Daydream™ shake – fructose	47.1 g powder 305.0 g water	742 [177.2] {1,567}	14.7 {31.3}	1.8 {3.9}	25.0 {53.0}	23.6 {50.0}	1.5 {3.1}
Lightly Salted Sunshine™ soy protein chips	48.1	808 [193.0] {1,672}	13.5 {28.0}	3.8 {8.0}	25.0 {52.0}	0.0 {0.0}	0.0 {0.0}
Soy spaghetti	42.4 (dry)	636 [151.9] {1,467}	10.6 {24.6}	1.1 {2.6}	25.0 {57.9}	1.5 {3.5}	0.8 {1.8}

^1^The amount of water listed for some products is the amount of water needed to prepare that product. This volume is in addition to the 250 g of water consumed with every product as part of the test methodology.

^2^The protein in all of the products except the spaghetti is from soy. For the spaghetti, one serving provided 14 grams of protein, 8 grams from soy and 6 grams from semolina. Each Chocolate Daydream™ shake contained 20 g soy protein. Each Peanut Butter Chocolate Pal™ bar contained 16 g soy protein. The soy protein chips contained 7 g soy protein per serving

Each portion of the reference food was prepared the day before required by dissolving the glucose in 250 g of water and storing overnight at 4°C. The individual portions of the test foods were prepared the day before required, except for the soy spaghetti. The individual portions of the uncooked soy spaghetti were weighed the day before required. On the testing day, each portion of dry spaghetti was cooked for 4 minutes in boiling water and drained. The reference and test foods were served with 250 g of plain water. The subjects consumed all the food and fluid served to them at a comfortable pace within 12 minutes.

### Experimental Procedures

The experimental methods used in the current study have been previously described [31] and are briefly outlined here. In both experiments, study subjects consumed the reference food on two separate occasions and each of the test foods on one occasion only after a 10-hour overnight fast. The reference food was consumed on the first and last test sessions, and test foods were consumed in random order in between. Each test session was completed on a separate morning with at least a day between subsequent sessions.

On each test day a baseline, finger-prick blood sample was obtained for blood glucose and plasma insulin determinations using an automatic, non-reusable lancet device (Safe-T-Pro^®^, Boehringer Mannheim GmbH, Mannheim, Germany). Following consumption of the reference or test food, additional blood samples were collected at 15, 30, 45, 60, 90 and 120 minutes. Blood glucose concentrations were measured immediately after the blood samples were collected. Blood samples collected for plasma insulin determination were centrifuged for 30 seconds immediately after collection and the plasma layer from each sample was transferred into a labeled, uncoated microcentrifuge tube and stored at -20°C until analyzed.

### Blood Glucose and Glycemic Index Determinations

The glucose concentration in each of the whole capillary blood samples was analyzed in duplicate using a glucose dehydrogenase/mutarotase enzymatic reaction using a HemoCue^® ^beta-glucose photometric analyzer (HemoCue AB, Ängelholm, Sweden). Duplicate readings were accepted if the two separate measurements for each time point were within 0.3 mmol/L of each other. If the readings were not within 0.3 mmol/L of each other, then an additional 2 blood glucose sample readings were taken from the subject within approximately 40 – 60 seconds after the initial readings. The two or three similar (i.e. within 0.3 mmol/L) readings were then averaged together to obtain the blood glucose response for that time point. A two-hour blood glucose response curve was constructed and the incremental area under the glucose response curve (IAUC) was calculated. The GI value for each test food was calculated for each subject by dividing the two-hour blood glucose IAUC value for the test foods by their average two-hour blood glucose IAUC value for the reference food and multiplying by 100 to obtain a percentage score. The final reported GI value for each test food is the mean GI value for that food in the group of 10 subjects.

### Plasma Insulin and Insulin Index Determinations

The concentration of insulin in each plasma sample was analyzed using a solid-phase antibody-coated tube radioimmunoassay kit (Diagnostic Products Corporation, Los Angeles, CA, USA). A two-hour plasma insulin response curve was constructed from the data and the IAUC of the insulin response curve was calculated. The II value for each test food was calculated for each subject by dividing their plasma insulin IAUC value for the test foods by their mean plasma insulin IAUC value for the reference food and multiplying by 100 to obtain a percentage score.

### Statistical Analyses

Sample size calculations (90% power, level of significance = 0.05) using data from published GI studies indicated that a minimum of eight study subjects would be needed to detect significant differences among the GI values of the reference and test foods. Analysis of variance (ANOVA) and the Fisher PLSD test for multiple comparisons were used to determine significant differences between the test foods' mean GI and II values. Statistical analyses were conducted using Statview Student™ software (version 4, Abacus Concepts Inc., Berkley, CA, USA). Significance was assumed at P < 0.05.

## Results

### Experiment 1: Low-Carbohydrate Soy Foods

**Figure 1:**
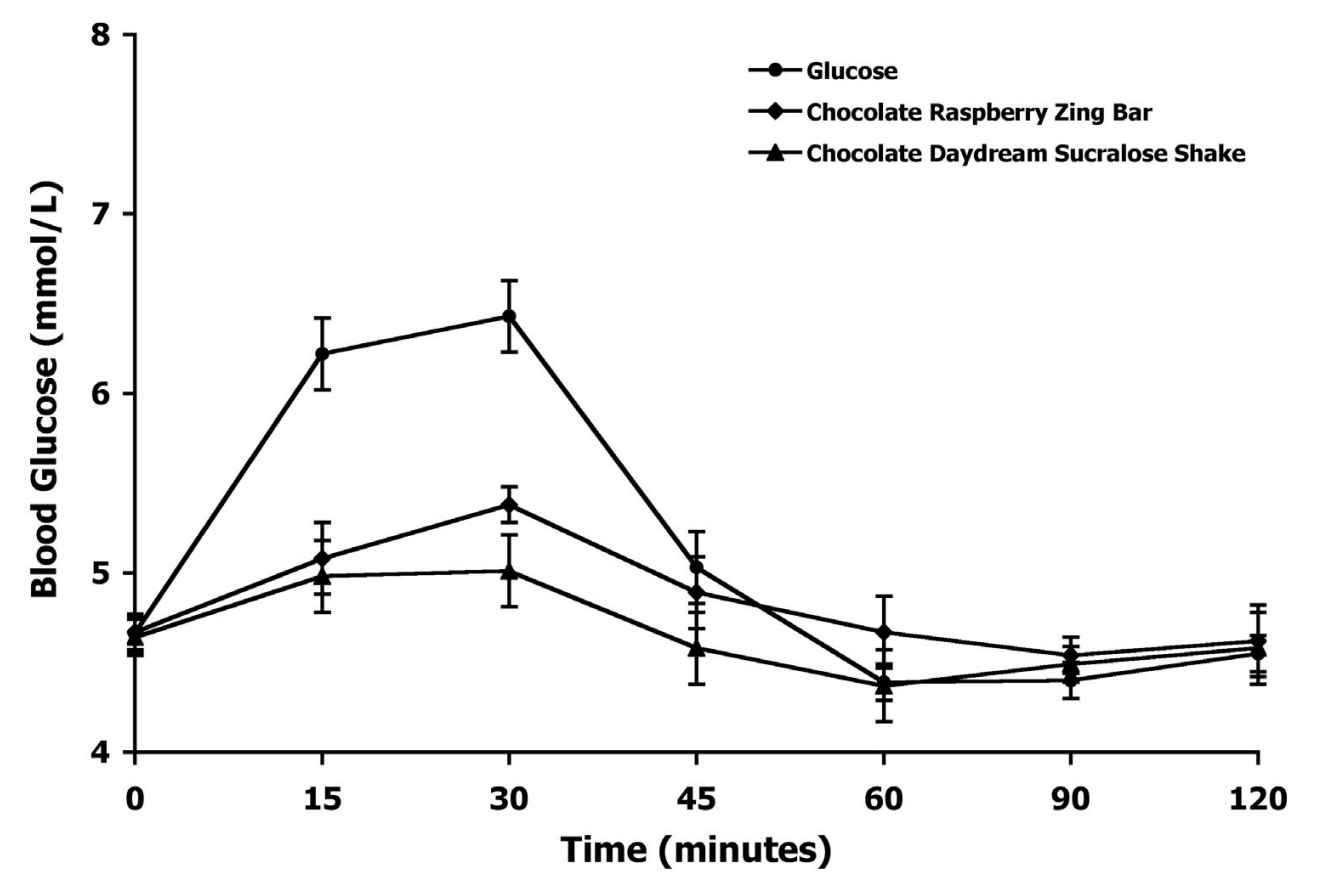
**Blood glucose response curves of low-carbohydrate soy products in Experiment 1**. The mean blood glucose response curves for the equal-carbohydrate portions of the reference food (glucose) and the two soy-based, low-carbohydrate food products tested in Experiment 1. Data are expressed as the change in blood glucose concentration from the fasting baseline concentration. Bars for each data point represent standard error of the means (SEM).

The mean blood glucose response curves for the reference and two test foods are shown in Figure 1. The reference food produced a much larger rise in blood glucose during the first 30 minutes and a greater overall glycemic response than the two test foods. The two test foods produced slightly different glucose response curves with the Chocolate Raspberry Zing™ bar producing a higher glycemic response than the Chocolate Daydream™ sucralose shake. However, both foods produced very low glycemic response curves.

**Figure 2:**
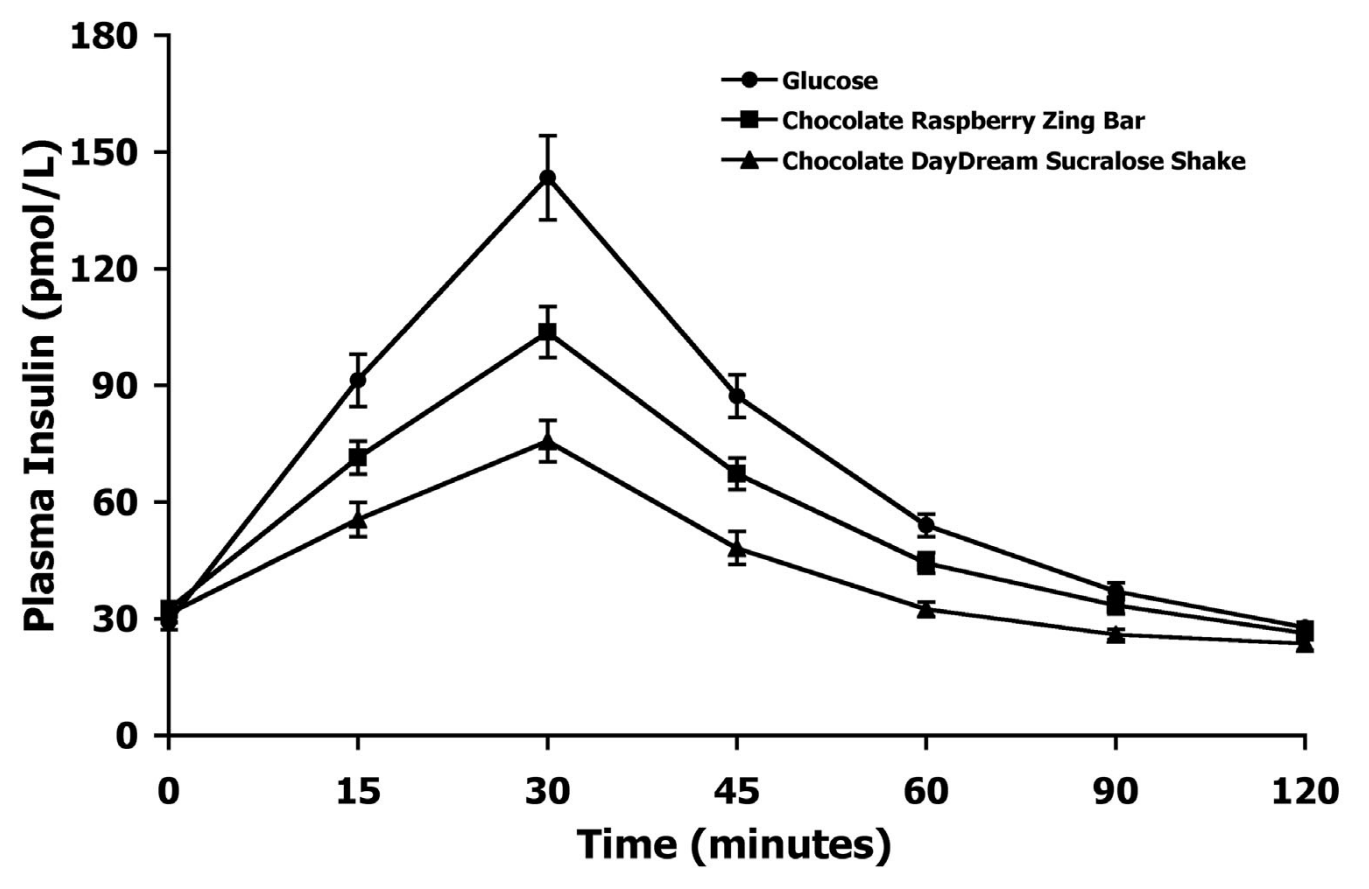
**Plasma insulin response curves of low-carbohydrate soy products in Experiment 1**.The mean plasma insulin response curves for the equal-carbohydrate portions of the reference food (glucose) and the two soy-based, low-carbohydrate food products tested in Experiment 1. Data are expressed as the change in plasma insulin concentration from the fasting baseline concentration. Bars for each data point represent standard error of the means (SEM).

The mean plasma insulin response curves for the reference and two test foods are shown in Figure 2. The plasma insulin responses observed for the reference food and the test foods showed a similar profile to their concurrent blood glucose responses. The reference food produced the highest peak plasma insulin concentration and the largest overall plasma insulin response, followed by the Chocolate Raspberry Zing™ bar and the Chocolate Daydream™ sucralose shake, respectively.

### Experiment 2: Other Soy Foods

**Figure 3:**
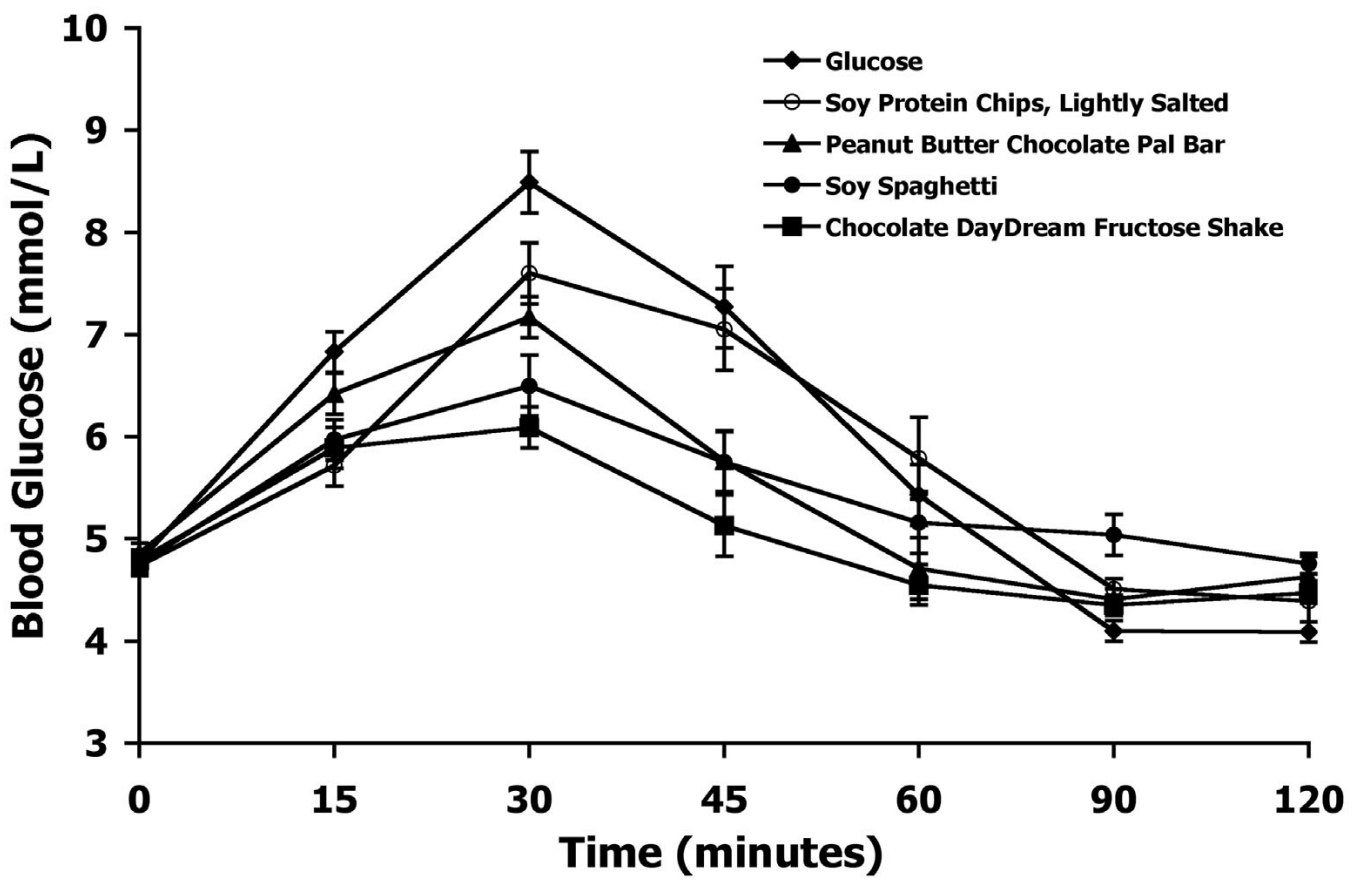
**Blood glucose response curves of soy products in Experiment 2**.The mean blood glucose response curves for the equal-carbohydrate portions of the reference food (glucose) and the four soy-based food products tested in Experiment 2. Data are expressed as the change in blood glucose concentration from the fasting baseline concentration. Bars for each data point represent standard error of the means (SEM).

The mean blood glucose response curves for the reference and the four test foods are shown in Figure 3. Similar to the results observed in Experiment 1, the reference food produced a large rise in blood glucose during the first 30 minutes and the greatest overall glycemic response. The four test foods varied in their peak blood glucose concentrations and their overall glycemic responses. Among the test foods, the baked soy protein chips produced the largest glycemic response followed by the Peanut Butter Chocolate Pal™ bar, the soy spaghetti, and the Chocolate Daydream™ fructose shake.

**Figure 4:**
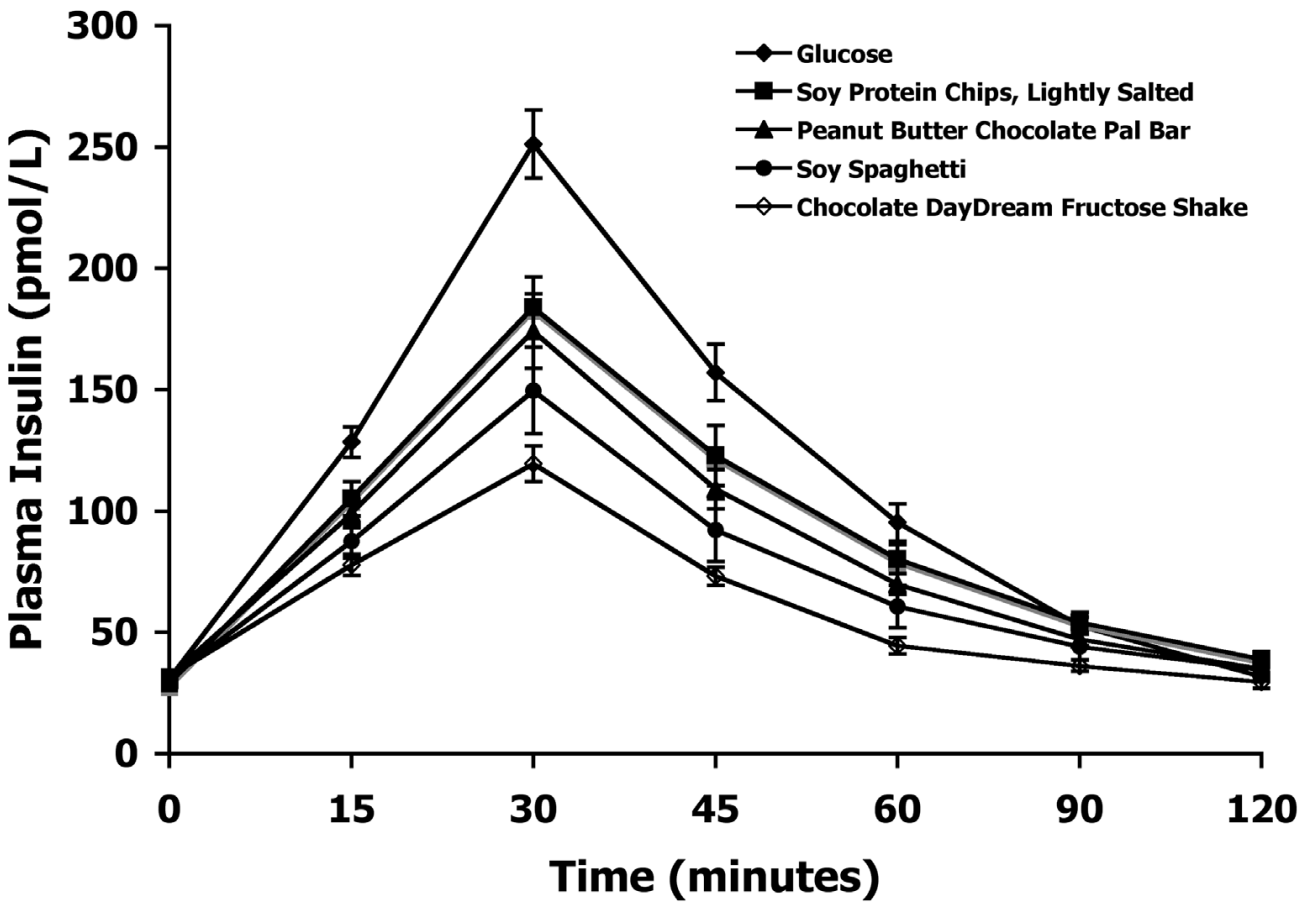
**Plasma insulin response curves of soy products in Experiment 2**.The mean plasma insulin response curves for the equal-carbohydrate portions of the reference food (glucose) and the four soy-based food products tested in Experiment 2. Data are expressed as the change in plasma insulin concentration from the fasting baseline concentration. Bars for each data point represent standard error of the means (SEM).

The mean plasma insulin response curves for the reference and the four test products are shown in Figure 4. The foods' average plasma insulin responses were similar to their respective mean plasma glucose responses. The reference food produced the largest plasma insulin response, followed by the four test foods in the same order as their glycemic responses.

### Glycemic and Insulin Indices

**Figure 5:**
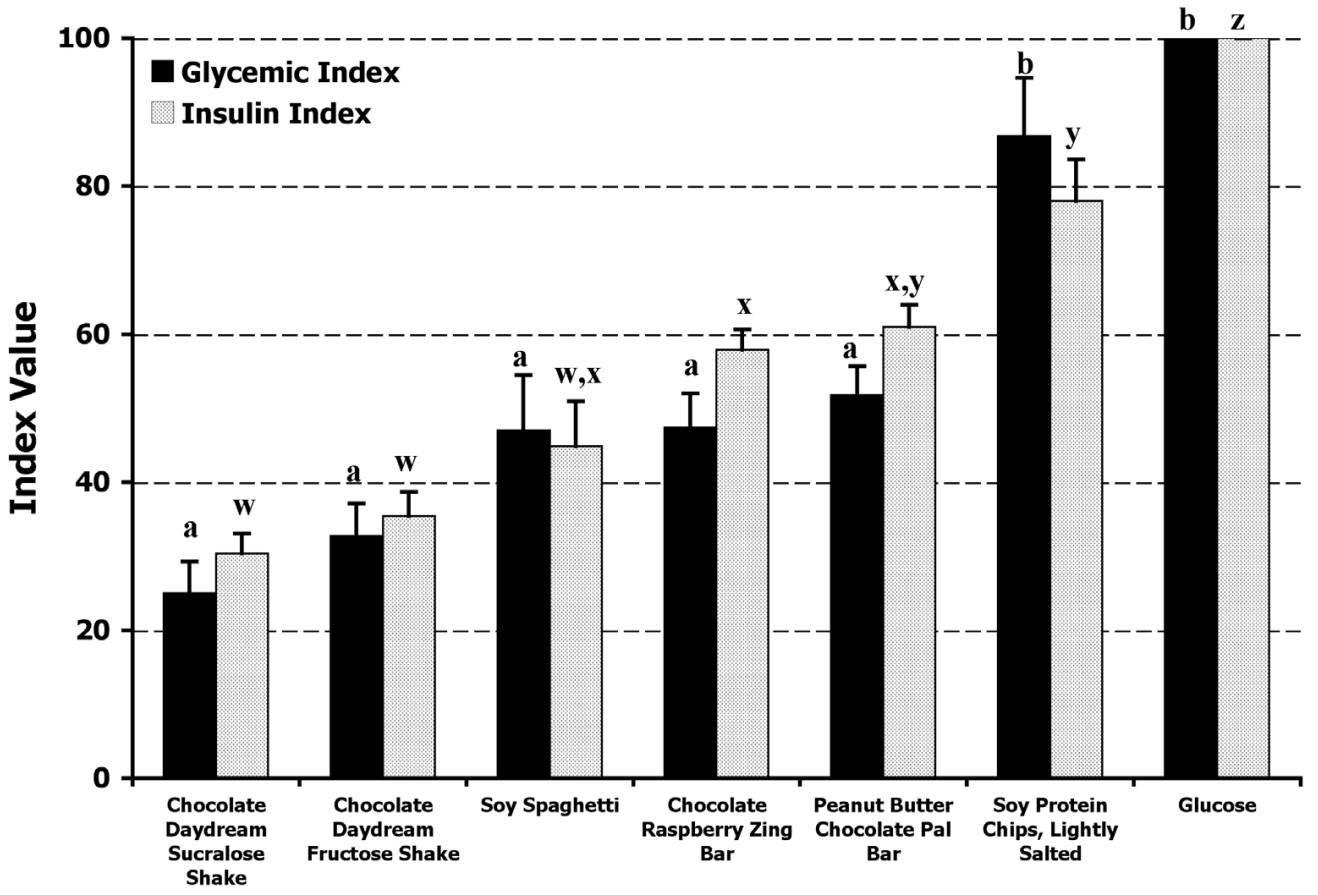
**Glycemic and insulin index values of tested soy products**. The mean (± SEM) glycemic index and insulin index for the reference food (glucose) and the six tested soy-based food products. The dark bars represent the glycemic index values and the light bars represent the insulin index values. For the GI values, columns with different superscripts (a, b) are significantly (P < 0.001) different. Columns representing the II values with different superscripts (w, x, y, z) are significantly different (P < 0.001).

The mean GI value of the glucose reference was significantly greater (P < 0.001) than the mean GI values of each of the test foods with the exception of the baked soy protein chips (Figure 5). The mean GI value of the soy protein chips was not different from the glucose reference, but was significantly greater (P < 0.001) than the mean GI values for the other five test foods. Despite a high GI value, the soy protein chips had only a medium GL value due to the small serving size and relatively low carbohydrate level (Table 3).

**Table 3:** Glycemic index and glycemic load^1 ^values for the six tested soy food products

		Glycemic Index			Glycemic Load	
**Test Food**	**Carbohydrate Tested (g)**	**Value ± SEM^2^**	**Category^3^**	**Carbohydrates/Serving (g)**	**Value**	**Category^4^**

Chocolate Daydream™ shake – sucralose	10.0	25.00 ± 4.28	Low	5	1.25	Low
Chocolate Daydream™ shake – fructose	25.0	32.73 ± 4.41	Low	34	11.13	Medium
Soy spaghetti	25.0	47.03 ± 7.48	Low	33	15.52	Medium
Chocolate Raspberry Zing™ bar	10.0	47.42 ± 4.55	Low	6	2.85	Low
Peanut Butter Chocolate Pal™ bar	25.0	51.82 ± 3.86	Low	31	16.06	Medium
Lightly Salted Sunshine™ soy protein chips	25.0	86.79 ± 7.86	High	13	11.28	Medium

^1^Glycemic Load = (GI × net carbohydrates)/100

^2^SEM = Standard Error of the Means

^3^Glycemic Index Category: Low = ≤ 55; Medium = 56 – 69; High = > 70 [54]

^4^Glycemic Load Category: Low = ≤ 10; Medium = 11 – 19; High = > 20 [54]

The mean II value of the glucose reference was significantly greater (P < 0.001) than the mean II values of each of the six test foods (Figure 5). The mean II of the soy protein chips was significantly higher (P < 0.001) than the mean II values for the soy spaghetti, Chocolate Daydream™ fructose shake, Chocolate Raspberry Zing™ bar, and the Chocolate Daydream™ sucralose shake. The mean II values for the Peanut Butter Chocolate Pal™ bar and the Chocolate Raspberry Zing™ bar were both significantly greater (P < 0.001) than the mean II values for the Chocolate Daydream™ fructose and Chocolate Daydream™ sucralose shakes.

## Discussion

The results of this study demonstrate that 5 of the 6 soy food products tested have a low GI value (GI ≤ 55). Of the 6 products tested, only the baked soy protein chips had a high GI value (GI > 70). However, when the amount of available carbohydrates in one serving of the soy protein chips was used to calculate a GL value, the soy protein chips had a medium GL value. The other products tested also were either low or medium GL foods.

An increasing body of evidence suggests that the GI and/or GL values of foods impact human health (see [5,6,12,32,33] for reviews). Low-GI diets have been shown to improve glycemic control in diabetic [14,15] and non-diabetic [16,17] subjects. Diet impacts the incidence of type 2 diabetes and the regulation of dietary carbohydrate has taken on a prominent role in dietary control of this chronic disease. Two recent meta-analyses [13,34] reported that consumption of low-GI foods rather than high-GI foods appears to modestly improve glycemic control by reducing plasma cholesterol, fructosamine, and hemoglobin A_1C _(Hb_A1c_) levels.

A number of studies suggest that high GI/GL diets may increase CVD risk [7-9], while several others indicate that low GI diets may reduce some CVD risk factors [9,14,15,20-23]. Meta-analysis results indicated that low GI diets significantly reduced total cholesterol (average reduction = 0.17 mmol/L; P = 0.03) and HbA_1c _(average reduction after 12 weeks = 0.45%; P = 0.02) compared to high GI diets [35]. These data suggest that dietary GI may improve some, but not all markers of cardiovascular disease risk.

Few studies have examined the effect of low GI diets on weight loss; however, there is some evidence that low GI diets may be beneficial [33]. In obese women, an energy-restricted, low GI diet significantly increased weight loss compared to an energy-restricted, high GI diet [18]. Spieth and co-workers [19] demonstrated that an *ad libitum *low GI diet significantly (P < 0.05) reduced BMI to a greater extent than did an energy-restricted, low fat diet. A recent study demonstrated that dietary GI was inversely associated with thigh intramuscular fat while GL was inversely associated with visceral abdominal fat in men [36]. Despite the potential benefits of low-GI diets on weight loss, a number of studies report no effect on weight loss [37,38].

Soy protein is a high quality protein that has been extensively studied. The quality of soy protein has been assessed through several metabolic studies of nitrogen balance [39-41], which have demonstrated that soy protein supports nitrogen balance on par with beef and milk proteins. One recent study reported that amino acids from soy protein appear in the serum sooner, but that this may lead to a more rapid breakdown of the amino acids in the liver [42].

Dietary soy consumption has been shown to have beneficial effects on several aspects of human health, including the diseases potentially influenced by dietary GI levels [43-45]. Soy consumption has been reported to modestly improve plasma lipid profiles [46,47], improve bone health [48], help reduce menopausal symptoms [49], and slightly reduce the risk of breast [50] and prostate cancers [51]. The health benefits of dietary soy have been attributed to its isoflavones as well as to the biological actions of its constituent proteins. However, an additional means of providing health benefits may be through the low GI of soy and soy foods.

The international table of GI and GL values [26] reports the GI/GL values of a number of soy foods. These values range from a low GI of 14 for soybeans canned in brine to a high GI of 115 for a tofu-based frozen dessert [24]. The Revival Soy^® ^products tested in this study fell within this range and with the exception of the baked soy protein chips were all within the low GI category. Only a few other studies have reported on the GI of soy-based foods. Packer et al. [25] indicated that gluten-free, soy-based, bread had a high GI value. In contrast, the addition of soy foods has been shown to lower the GI value of mixed meals [27]. Similarly, the replacement of unrefined wheat flour with soy flour lowers the GI value of parantha, an Indian snack food [52]. Similar to the Revival Soy^® ^bars tested in the current study, other snack bars containing soy have been shown to have low to medium GI values [53]. Previous data report that the GI values of spaghetti ranges from 27 – 68. The Revival Soy^® ^thin spaghetti had a GI value = 47, similar to other spaghetti products. Overall, these studies indicate that soy-based foods generally have a low to medium GI value and would be suitable for individuals concerned with regulating blood glucose and insulin levels.

The ingredients and form of a food product affect its GI value. For example, while soybeans have a low GI value, the use of high GI ingredients in soy foods can increase the GI value of the final product. This was likely the case with the baked soy protein chips. The baked soy protein chips contain potato starch and potatoes have a high GI value [33]. Additionally, the baked soy protein chips have a puffed physical form, which may lead to high GI values. Similar to planning diets with types of fat and protein in mind, types of carbohydrates should also be considered since carbohydrate types influence the GI. The substitution of high GI ingredients with low GI ingredients in food products like the baked soy protein chips may help keep the final GI value down.

In addition to the effects of form and content of foods on the GI value, consumption of other foods with low GI foods can affect the overall GI value of meal. Sugiyama et al. [34] demonstrated that adding soybean products (miso, natto, and ground soybean) lowered the GI of white rice by 20 – 40%. However, further studies are required before any conclusions can be drawn.

## Conclusion

With the apparent resurgence of interest in low-GI diets for weight loss and health benefits, it is important that information on the GI value of foods is available. Therefore, we conducted the current study to determine the GI value of a small variety of commercially available soy foods. The results of the current study demonstrate that soy food products generally have low GI values and low to medium GL values. Improvements in ingredient selection and usage may further improve glycemic responses to soy foods. The low GI of soy foods appears to be an additional benefit of soy for human health and suggests that soy foods are an appropriate part of diet plans intended to improve control over blood glucose and insulin levels.

## Competing interests

Robert M. Blair is the Research Manager of Physicians Pharmaceuticals, Inc. E. C. Henley is a consultant to Physicians Pharmaceuticals, Inc. Aaron Tabor is the CEO and Medical Research Director of Physicians Pharmaceuticals, Inc.

## Authors' contributions

RMB prepared the manuscript. ECH and AT participated in the coordination and design of the study and assisted with manuscript preparation.
